# The STAT3‐miR‐223‐TGFBR3/HMGCS1 axis modulates the progression of cervical carcinoma

**DOI:** 10.1002/1878-0261.12737

**Published:** 2020-07-01

**Authors:** Ju Zhang, Ming Jiang, Lili Qian, Xiao Lin, Weiguo Song, Yunfeng Gao, Ying Zhou

**Affiliations:** ^1^ Department of Laboratory Medicine The First Affiliated Hospital of USTC Division of Life Sciences and Medicine University of Science and Technology of China Hefei China; ^2^ School of Life Science University of Science and Technology of China Hefei China; ^3^ China‐US (Henan) Hormel Cancer Institute Zhengzhou China; ^4^ Department of Obstetrics and Gynecology The First Affiliated Hospital of University of Science & Technology of China Anhui Provincial Hospital Hefei China; ^5^ Department of Gynaecology and Obstetrics The Second Hospital of Anhui Medical University Hefei China

**Keywords:** cervical cancer, HMGCS1, miR‐223, TGFBR3

## Abstract

Cervical cancer is induced by persistent infections with high‐risk human papillomaviruses (HPVs), which produce the early protein 6 of HPVs (E6)/E7 protein that is involved in cell transformation by interacting with several oncoproteins or tumor suppressors. However, the role of noncoding RNA in mediating the pathogenesis of cervical cancer remains unclear. Here, we report that the novel signal transducer and activator of transcription 3 (STAT3)‐microRNA‐223‐3p (miR‐223)‐TGFBR3/HMGCS1 axis regulated by the E6 protein controls cervical carcinogenesis. miR‐223 was highly expressed in cervical tumor tissues, whereas TGFBR3 or HMGCS1 was significantly downregulated. miR‐223 targeted the 3′‐UTRs of TGFBR3 and HMGCS1 and suppressed their expression, leading to increased anchorage‐independent growth and cervical squamous cell carcinoma (CSCC) tumor growth *in vitro* and *in vivo*. The increased expression of miR‐223 was mediated by the transcription factor STAT3, whose activity was enhanced by E6 in the context of interleukin (IL)‐6 stimulation. In addition, exosomal miR‐223 derived from CSCC cells induced IL‐6 secretion by monocyte/macrophage in a coculture system *in vitro*, and IL‐6 secretion, in turn, led to enhanced STAT3 activity in CSSC cells, forming a positive feedback loop. Furthermore, modified miR‐223 inhibitor effectively suppressed tumor growth in cell line‐derived xenograft model, suggesting that miR‐223 is a potential promising therapeutic target in CSCC. In conclusion, our results demonstrate that the STAT3‐miR‐223‐HMGCS1/TGFBR3 axis functions as a key signaling pathway in cervical cancer progression and provides a new therapeutic target.

## Graphical abstract

Human papillomaviruses (HPVs) produce early protein 6 of HPVs protein in infected female cervical tissue cells to enhance signal transducer and activator of transcription 3 (STAT3) activation, which promotes microRNA‐223‐3p (miR‐223) transcription, leading to normal tissue to cervical squamous cancer transformation. Mechanistically, miR‐223 represses TGFBR3 or HMGCS1 at post‐transcriptional level. In tumor loci, exosomal miR‐223 derived from cervical cancer cells induces interleukin‐6 secretion of monocyte/macrophage, which, in turn, stimulates STAT3 signals.

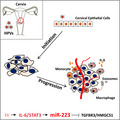

**Abbreviations**
CDX: cell line‐derived xenograft
CSCC: cervical squamous cell carcinoma
E6: early protein 6 of HPVs
GEO: Gene Expression Omnibus
HMGCS1: 3‐hydroxy‐3‐methylglutaryl‐CoA synthase 1
HPVs: human papillomaviruses
IHC: immunohistochemistry
IL‐6: interleukin 6
miR‐223: microRNA‐223‐3p
shRNA: short hairpin RNA
STAT3: signal transducer and activator of transcription 3
TCGA: The Cancer Genome Atlas Program
TGFBR3: transforming growth factor beta receptor 3

## 1 Introduction

There are over half million new cases of cervical cancer each year, making cervical cancer the fourth most commonly diagnosed cancer in females worldwide, and the diagnosis rate of cervical cancer is higher than that of any other gynecological tumor (Crosbie *et al*., 2013). High‐risk human papillomaviruses (HPVs), including the HPV16 and HPV18 subtypes, are considered the most important carcinogenic factors for cervical cell transformation (Chen *et al*., 2015). The early protein 6 of HPVs (E6)/E7 protein produced by HPVs has been reported to play a critical role in cervical cancer development. For example, E6 interacts with E6‐AP, forming the E3 enzyme for p53 ubiquitination and leading to the degradation of p53 (Martinez‐Zapien *et al*., 2016). This process leads to unscheduled cell cycle progression mediated by hyperactivated E2F transcription factors. In addition, carcinogenic HPVs also affect additional cancer‐related pathways, including the PI3K/AKT, signal transducer and activator of transcription 3 (STAT3), and metabolism pathways (Menges *et al*., 2006; Morgan and Macdonald, 2019; Munger *et al*., 2004). For the JAK2/STAT3 pathways, many studies have documented the aberrant activation of STAT3 in HPV‐positive cervical squamous cell carcinoma (CSCC) cells and that this activation is strongly associated with poor prognosis. For instance, the Rac1‐NF‐κB‐interleukin (IL)‐6 signaling axis regulates autocrine STAT3 activation resulting in cervical cancer development. Another report shows the specific region of the HPV16 LCR harboring a potential STAT3 binding site that regulates expression of viral oncogenes, thus suggesting a plausible productive interaction between HPV proliferation and STAT3 activation (Shukla *et al*., 2013). Similarly, another report shows that E6‐mediated STAT3 activation is essential for the HPV18 life cycle (Morgan *et al*., 2018). In particular, IL‐6 is well known as a critical regulator of STAT3 activation in most kinds of cancers. In cervical cancer, IL‐6 is reported to be highly upregulated and strongly associated with a poor outcome (Johnson *et al*., 2018). Although the autocrine IL‐6 regulation of cancer progression is well documented, the paracrine effects of IL‐6 secreted from stromal cells or immune cells on cancer development have also been reported (Grivennikov and Karin, 2008; Morgan and Macdonald, 2019; Yu *et al*., 2015). However, whether the latter mechanism also contributes to cervical cancer progression needs to be investigated. In addition to cytokines, many reports have shown that exosomes containing a large number of regulators are another modulator of cancer progression (Azmi *et al*., 2013). Of these regulators, exosomal microRNAs secreted from stromal cells, immune cells, or cancer cells have potent effects on tumor initiation and development (Fang *et al*., 2018; Kobayashi *et al*., 2014).

miRNAs are a class of evolutionarily conserved, regulatory, noncoding RNAs, that are approximately 21–23 nucleotides in length and have broad impacts on biology, including impacts on development, differentiation, chronic diseases, and cancer (Ha and Kim, 2014; Treiber *et al*., 2019). miRNAs regulate the stability and translation efficiency of target mRNAs by binding to their 3′‐UTRs. A variety of miRNAs, including miR‐19a/b, miR‐125b, miR‐21, and miR‐7, have been reported to be dysregulated in cervical cancer (Fan *et al*., 2016; Liu *et al*., 2013a; Ribeiro *et al*., 2015; Wang *et al*., 2019; Xu *et al*., 2012). microRNA‐223‐3p (miR‐223) has been indicated to be overexpressed in cervical cancer or HPV‐transformed raft tissue (Wang *et al*., 2008). However, the functional role of miR‐223 in cervical carcinogenesis has not been described.

Transforming growth factor beta receptor 3 (TGFBR3), also known as betaglycan, is an accessory coreceptor of TGF‐β (Johnson *et al*., 1995). Increasing evidence has shown that the function of TGFBR3 is complex and that TGFBR3 may inhibit or promote TGF‐β signaling in the context of different cells (Li *et al*., 2018; Liu *et al*., 2013b). Recently, TGFBR3 was shown to exhibit antifibrotic properties in the heart and lung, suggesting that it is a potent TGF‐β‐neutralizing agent (Eickelberg *et al*., 2002). Additionally, decreased TGFBR3 expression enhances the metastatic ability and tumor growth of renal cell carcinoma cells suggesting that TGFBR3 mainly acts as a suppressor of cancer development (Nishida *et al*., 2018). 3‐hydroxy‐3‐methylglutaryl‐CoA synthase 1 (HMGCS1) is a metabolic enzyme that condenses acetyl‐CoA with acetoacetyl‐CoA to form HMG‐CoA which is an important substrate in the mevalonate pathway. To date, only a few studies have investigated the role of HMGCS1 in cancer development. The knockdown of HMGCS1 results in more attenuated cell proliferation rates in BRAF^V600D/E^‐expressing melanoma cells (Zhao *et al*., 2017). HMGCS1 is expressed at a low level in cervical cancer based on analysis of five independent transcriptomic Gene Expression Omnibus (GEO) datasets suggesting that HMGCS1 may play another role in cancer progression (Kori and Yalcin Arga, 2018).

In the present study, we found that miR‐233 was obviously upregulated in squamous cervical cancer tissues, and controlled the development and progression of CSCC both *in vitro* and *in vivo*, suggesting that miR‐223 plays a tumor‐promoting role in CSCC cells. Furthermore, we demonstrated the mechanism by which miR‐223 was directly regulated by STAT3 at the transcriptional level and showed that TGFBR3 and HMGCS1 were targeted. Additionally, we found CSCC cell‐derived exosomes containing high levels of miR‐223 that induced IL‐6 secretion from monocytes/macrophages *in vitro*. *In vivo*, monocyte promoted CSCC cell growth in the cell line‐derived xenograft (CDX) model, and a miR‐223 inhibitor significantly suppressed tumor growth. The newly identified STAT3‐miR‐223‐TGFBR3/HMGCS1 axis functions as a key pathway that modulates CSCC tumorigenesis.

## 2 Materials and methods

### 2.1 Tissue samples, immunohistochemical staining, and H&E staining

Fresh human surgical specimens containing adjacent and tumor tissues were obtained from 50 patients undergoing surgery for CSCC at First Affiliated Hospital of Science and Technology of China, Hefei, China. All the patients underwent a tumorectomy prior to the receipt of any adjunctive therapy. The tissue collection and analyses were approved by the Ethics Committee of First Affiliated Hospital of Science and Technology of China, and all the participants provided written informed consent. The studies were performed in accordance with the International Ethical Guidelines for Biomedical Research Involving Human Subjects (CIOMS) and the intentions of the Declaration of Helsinki. H&E and immunohistochemistry (IHC) staining were performed using paraffin‐embedded sections of biopsies from CSCC samples according to standard protocols by Abcam (Cambridge, MA, USA). The antibodies used for IHC were as follows: anti‐HMGCS1 (ab194971; Abcam), anti‐TGFBR3 (#2519; CST, Danvers, MA, USA), anti‐VEGFA (ab1316; Abcam), anti‐CD34 (ab81289; Abcam), and anti‐Ki67 (ab16667; Abcam). Finally, the adjacent and tumor sections of each sample were assessed according to staining intensities.

### 2.2 Cell lines

The human squamous cervical carcinoma cell lines SiHa (HPV16+) and ME180 (HPV18+) were purchased from the Shanghai Institute of Cell Biology, Chinese Academy of Sciences (Shanghai, China). The cells were cultured in RPMI‐1640 medium (BI, Kibbutz Beit HaEmek, Western Galilee area of Northern Israel) supplemented with 10% FBS (Gibco, Grand Island, NY, USA) and penicillin G (100 U·mL^−1^) and streptomycin (0.1 mg·mL^−1^; Solarbio, Beijing, China). 293T cells (ATCC, Manassas, VA, USA) were maintained in Dulbecco's Modified Eagle's medium (BI). The cells were plated in 5% CO_2_ and at 37 °C under humidity condition. All cell lines were confirmed to be mycoplasma‐free and authenticated by PCR.

### 2.3 Reagents, plasmids, and the establishment of stable transfection cells

A precursor of human miR‐223 was amplified from human genomic DNA (extracted from SiHa cells) with the following specific primers: forward: 5′–3′ ACTCTGTTAATCAGCTTGCTT; and reverse: 5′–3′ ATAATGCCTCAGTTCCTGCAAAG. The 400‐bp product was digested with EcoRI and BamHI (Takara, Tokyo, Japan) and then was introduced into the pCDH expression vector (pCDH‐CMV‐MCS‐EF1‐GFP‐T2A‐Puro). The constructed plasmids were sequenced for authentication. MiR‐223 sponge vector which has 4 repeat tandem complementary sequences of mature miR‐223 was purchased from GenePharma Co., Ltd (Shanghai, China). STAT3 overexpression vector (pCMV‐STAT3) was purchased from Youbio (Changsha, China). The HPV16 E6 overexpression plasmid was gift from Ying Zhou (the First Affiliated Hospital of Science and Technology of China). The oligos for short hairpin RNA (shRNA) targeting E6, STAT3, TGFBR3, and the HMGCS1 were synthesized by Sangon Biotech (Shanghai, China). To prepare the lentivirus, the packaging plasmids (psPAX2 and pMD2.G) were cotransfected with pCDH‐pre‐miR‐223 with ratio of a 1 : 1 : 1 into 293T cells. After 48 h, the supernatant was collected and filtered by using a 0.45‐μm syringe filter. The lentiviral supernatant was mixed with fresh medium at a 1 : 1 ratio and was added into six‐well plates with 80% confluent cells. Polybrene (8 μg·mL^−1^) was used to increase the efficiency of transfection. After 12 h, complete medium with 3 μg·mL^−1^ puromycin was used to select the positive clones. The STAT3 inhibitor (C188‐9) was purchased from MCE (Monmouth Junction, NJ, USA). The human cytokine IL‐6 was obtained from R&D (Minneapolis, MN, USA).

### 2.4 Cell line‐derived xenograft model

Six‐week‐old nude mice were purchased from the Model Animal Research Center of Nanjing University (Nanjing, China) and maintained under specific pathogen‐free conditions at the University of Science and Technology of China (USTC) and were used for the tumor formation assay. A total of 2 × 10^6^ overexpression cells, knockdown cells, or the corresponding control cells were resuspended in 100 μL of a 1 : 1 mixture of PBS and Matrigel and were then subcutaneously injected into the right flanks of nude mice. Tumor progression was monitored by measuring the tumor volume which was calculated as (0.5 × width) × (height^2^). After 4 weeks, the mice were sacrificed, and the tumors were analyzed by IHC, quantitative PCR (qPCR), and western blotting. For therapeutic model, cholesterol‐conjugated miR‐223 inhibitors were synthesized by GenePharma Co., Ltd. Two weeks after implantation, cholesterol‐conjugated inhibitor or control was delivered *via* the tail vein every 4 days for 16 days. Then, the mice were sacrificed and the tumors were measured. All the animal studies were conducted according to protocols approved by the Animal Ethics Committee of First Affiliated Hospital of USTC.

### 2.5 Luciferase assay

The distal promoter region of miR‐223 was amplified from human genomic DNA according to the method described by Fukao *et al*. (2007). The amplified fragment was digested by SacI and XhoI and was introduced into pGL4.2[*lun2*/Puro] (Promega, Madison, WI, USA). The 3′‐UTRs of human *TGFBR3* and *HMGCS1* were cloned in between the SacI and XhoI sites of pmir‐GLO (Promega) using PCR‐generated fragments. The mutant construct was generated by PCR‐directed mutagenesis using Fast Mutagenesis Kit (TransGen, Beijing, China). SiHa and 293T cells were seeded onto 48‐well plates (~ 1 × 10^5^ cells per well) the day before transfections were conducted. pGL4.2‐miR‐223 (100 ng·well^−1^) and pRL‐TK (50 ng·mL^−1^) reporters were cotransfected into SiHa/shSTAT3, SiHa/sh16E6, and the corresponding control cells. Forty‐eight hours later, the luciferase activity was determined using a dual‐luciferase reporter kit (Promega). For the 3′‐UTR assay, miR‐223 overexpression, knockdown plasmids, or control plasmids were cotransfected with pmir‐GLO vector or mutant vectors into 293T cells. Forty‐eight hours later, the luciferase activity was determined by using a dual‐luciferase reporter kit (Promega).

### 2.6 RNA extraction and real‐time qPCR

Total RNA was isolated from tissues and cells with TRIzol reagent (Invitrogen, Carlsbad, CA, USA) according to the manufacturer's protocol. Reverse transcription and qPCR for miRNAs or mRNA were conducted as described previously. Briefly, qPCR for miR‐223 or HMGCS1 and TGFBR3 mRNA was performed using a SYBR premix ExTaq Reverse Transcription PCR Kit (Takara), and the expression levels were normalized to those of GAPDH or U6. The sequences of the primers are listed in Table S1. All the reactions were performed in triplicate.

### 2.7 Western blot analyses

The tissues and cells were lysed in RIPA buffer. The protein concentration was determined by Protein Quantification Kit (BCA Assay; Thermo, Waltham, MA, USA). The protein levels were analyzed *via* western blotting and performed as described as previously. The antibodies were as follows: anti‐STAT3 antibody (#9139; CST), anti‐Flag antibody (#F1804; Sigma, St. Louis, MO, USA), anti‐STAT3(705) antibody (#9145; CST), anti‐STAT3(727) antibody (#9134; CST), anti‐Actin antibody (#KM9001T, SUNGENE Biotech, Tianjin, China), anti‐TGFBR3 antibody (#2519; CST), and anti‐HMGCS1 antibody (Ab194971; Abcam). The protein levels were normalized to those of β‐Actin.

### 2.8 ChIP assay

ChIP assays were performed with ChIP Assay Kit (Abcam) according to the manufacturer's protocol. Briefly, the crosslinking of proteins to DNA was accomplished by the addition of 1% formaldehyde for 15 min to cultured cells at room temperature. After sonication, the chromatin was immunoprecipitated with 4 μg of anti‐STAT3, anti‐histone H3, or anti‐IgG (Abcam) antibodies at 4 °C overnight. PCR was performed by using specific primers as described in Table S1.

### 2.9 Exosome isolation and identification

Exosome isolation and identification were performed as described previously (Zhou *et al*., 2019). In brief, a total of 10 mL cell conditioned medium was mixed with ExoQuick exosome precipitation solution. After incubation overnight, the mixture was centrifuged at 2000 ***g*** for 30 min at 4 °C. The pelleted exosomes were subjected to protein assay, RNA extraction, or *in vitro* treatment. For the protein assay, the exosome preparations used BCA Protein Assay Kit (Thermo). For RNA extraction from exosomes, miRNeasy Mini Kit (Qiagen) was used. For *in vitro* treatment, 50 μL PBS containing 5 μg of exosomes was added to recipient cells for 48 h.

### 2.10 Statistical analysis

Each experiment was performed at least three times. The results are presented as the mean ± the standard deviation (SD). Statistical significance was evaluated by an unpaired Student's *t*‐test in GRAPHPAD PRISM (La Jolla, CA, USA). Statistically significant changes (*P < 0.05; **P < 0.01; and ***P < 0.001) are indicated.

## 3 Results

### 3.1 Upregulation of miR‐223 correlates with advanced stages of CSCC

**Fig. 1:**
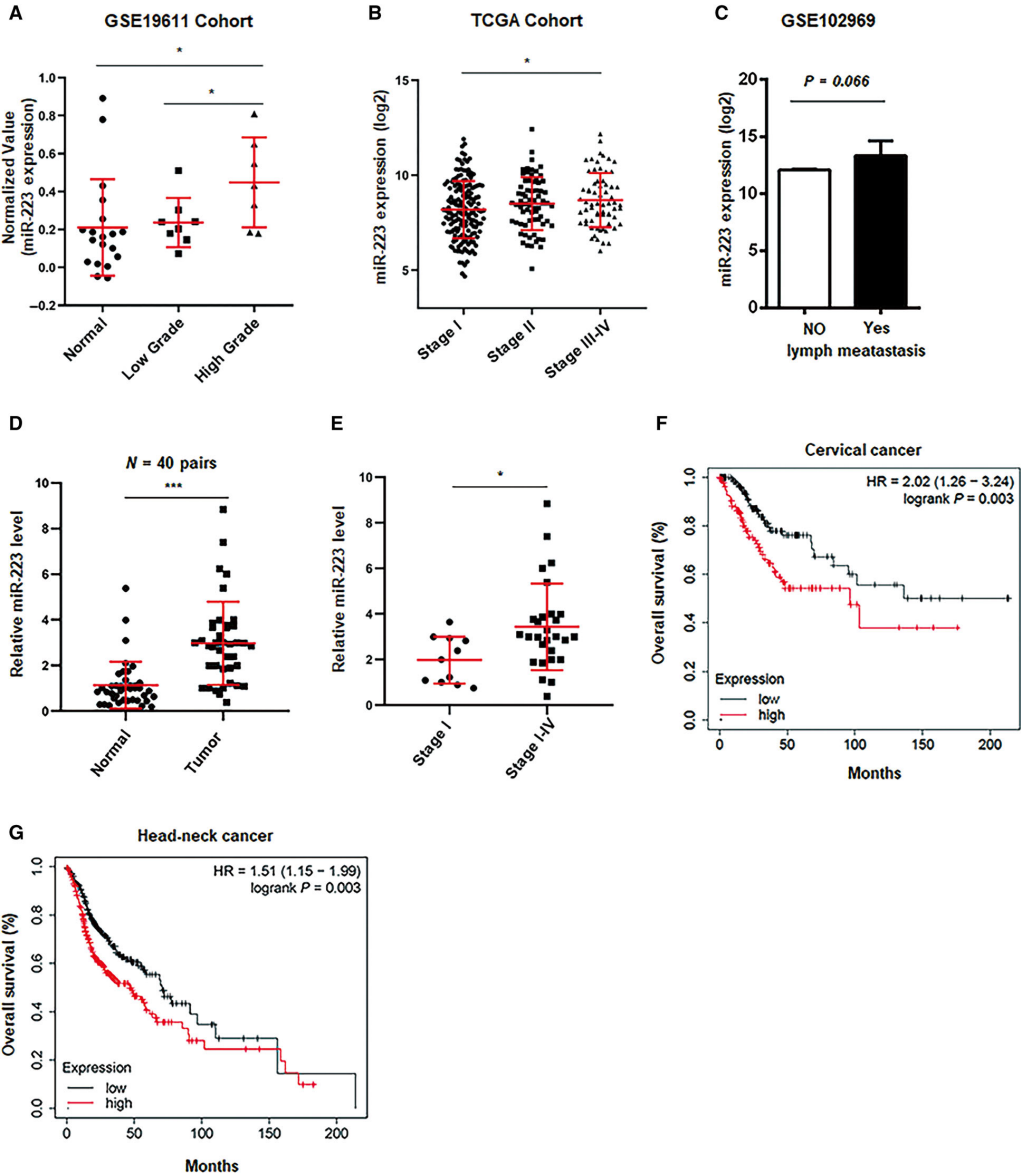
Upregulated of miR‐223 is associated with advanced stage of CSCC. (A, B) Relative miR‐223 expression was analyzed in different stages of cervical cancer in GES19611 cohort (A) and TCGA cohort (B). (C) miR‐223 value was analyzed in metastasis or nonmetastasis group in GSE102969 cohort. (D) miR‐223 level was examined by qPCR in cervical cancer tissues and normal tissues (*n* = 40 pairs). (E) miR‐223 level was determined in indicated stages of cervical cancer tissues. In (D) and (E), bars were representative of three independent experiments. (F, G) The overall survival rate was calculated in miR‐223‐high or miR‐223‐low group in cervical cancer (F) and head–neck cancer (G) based on TCGA database. *P < 0.05; ***P < 0.001.

To demonstrate the clinical significance of miR‐223 expression in CSCCs, we first investigated whether miR‐223 was expressed at different levels in different stages of cervical cancer based on the open‐access database [(GEO and The Cancer Genome Atlas Program (TCGA)]. As shown in Fig. 1A,B, higher levels of miR‐223 were observed in advanced stages of CSCC. In addition, we also found that the miR‐223 level was slightly upregulated in the lymphatic metastasis group (Fig. 1C). These results suggested that a high level of miR‐223 was associated with poor prognosis in CSCC. To confirm these results in clinical samples, we examined miR‐223 expression in clinical CSCC tissues. As shown in Fig. 1D, mature miR‐223 expression was obviously upregulated in tumor tissues compared with adjacent tissues. Consistent the observation above, miR‐223 was upregulated in clinical samples of advanced stages (Fig. 1E). Additionally, we found that high expression of miR‐223 was associated with worse outcomes in CSCC patients based on TCGA database (Fig. 1F). We also found that elevated miR‐223 was associated with poor prognosis of head–neck squamous carcinoma, which is another HPV^+^ cancer type (Fig. 1G). Collectively, these results demonstrated that miR‐223 is highly expressed in cervical cancer and functions as a potential biomarker of poor prognosis in CSCC.

### 3.2 miR‐223 promotes CSCC progression

**Fig. 2:**
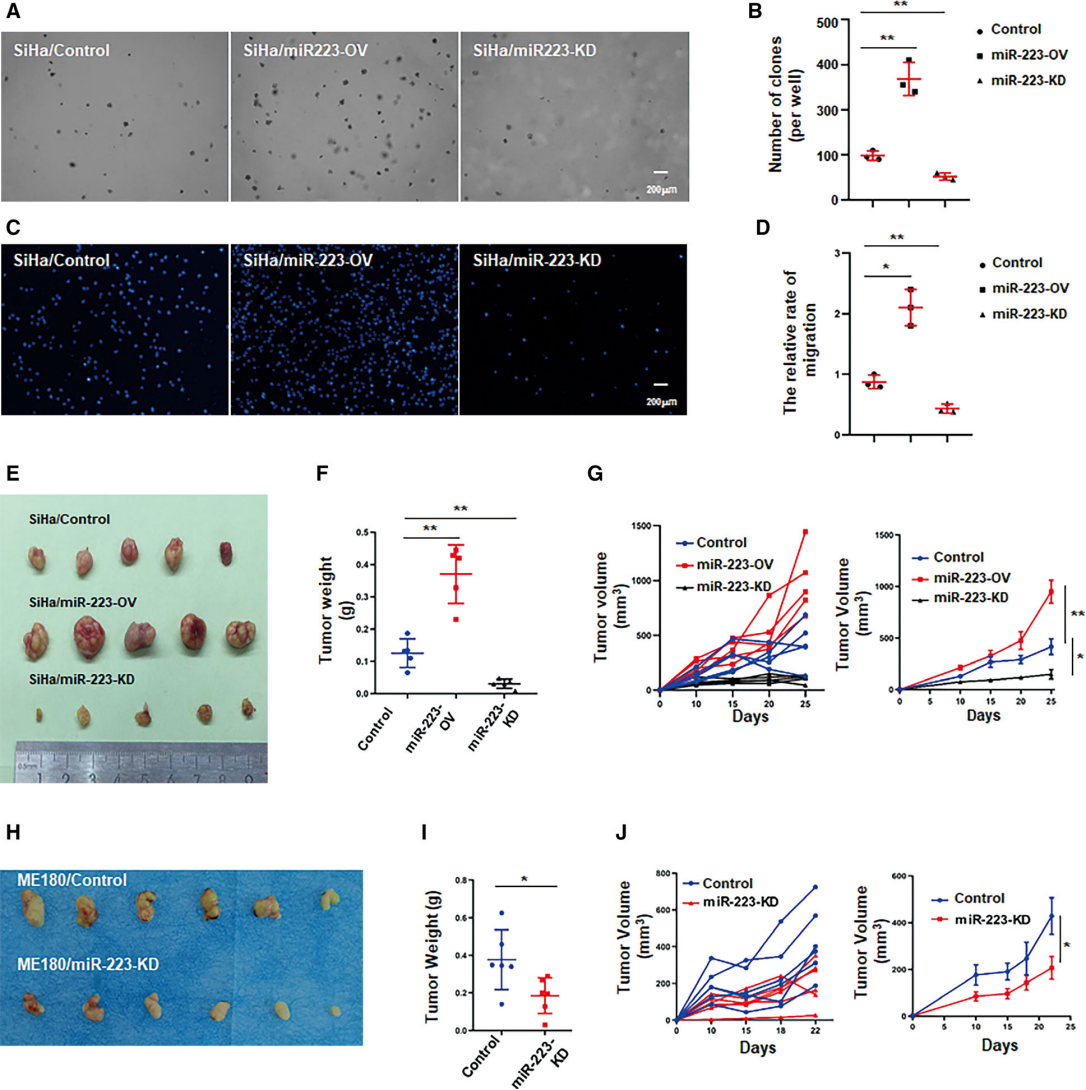
miR‐223 positively regulates cervical cancer development *in vitro* or *in vivo*. (A) Representative images of the colony formation of modified SiHa cells by using anchor‐independent growth (soft agar) assay. In brief, a total of 8 × 10^3^ cells were seeded into six‐well plates with 0.4% agar (upper layer). Mixture was placed in 5% CO_2_ and at 37 °C in a humidity condition for 3 weeks. The colonies were counted by inverted microscope. (B) The statistics of colony number in (A). (C) Representative images of the migration of modified SiHa cells by using Transwell assay. Mircrographs were shown at original magnifications (200 μm) as indicated. (D) The statistic of relative migration index (normalized with migrated control cells) in (C). (E) Representative images of tumors of indicated groups. (F) Quantitative analysis of tumor weights in (E). (G) Time course of tumor growth in implanted mice and tumor volume was measured every 5 days for 30 days after implantation. Left was individual and right was average of group. (H) Representative images of tumor from mice of each groups (ME180‐CDX model). (I) Quantitative analysis of tumor weights in (H). (J) Time course of tumor growth in implanted mice and tumor volume was measured every 5 days for 30 days after implantation. These results represent one of three independent experiments. Data were statistically analyzed with Student's *t*‐test, and value was shown as mean ± SD. *P < 0.05, **P < 0.01, NS = not significant.

To investigate the role of miR‐223 in CSCC development, we established stable cell lines in which miR‐223 was overexpressed or knocked down (designated SiHa/miR‐223‐OV, SiHa/miR‐223‐KD, ME180/miR‐223‐OV, ME180/miR‐223‐KD; Fig. S2A). The knockdown or overexpression efficiency was examined in corresponding cell lines by using qPCR assays (Fig. S2B). To evaluate whether miR‐223 affected the colony formation ability of CSCC cells, we seeded the same number of miR‐223‐OV, miR‐223‐KD, or control cells into 6‐well plates coated with the indicated concentration of soft agar. After 3 weeks, the colonies were counted using microscope. We found that miR‐223 overexpression significantly increased the colony number, whereas knockdown of miR‐223 obviously inhibited colony formation (Fig. 2A,B). Enhanced migration ability is one of the hallmarks of malignant tumors (Hanahan and Weinberg, 2011). Here, we used a Transwell assay to investigate the role of miR‐223 in CSCC cell migration. We found that miR‐223 overexpression promoted CSCC cell migration, whereas lower migration activity was observed in knockdown cells (Fig. 2C,D). Furthermore, to explore whether miR‐223 was involved in tumor growth *in vivo*, a CDX model was established by injecting the modified cells into the flanks of nude mice. We found that miR‐223 significantly promoted tumor growth in the nude mice, whereas the knockdown of miR‐223 decreased tumor volume and weight (Fig. 2E–G and Fig. S2C). Similar results were obtained from the CDX model in which ME180 cells with miR‐223 alterations were injected into nude mice (Fig. 2H–J and Fig. S2F). The IHC assays indicated that the levels of Ki67 and VEGFA were obviously increased in the miR‐223‐OV tumor tissues and were downregulated in the miR‐223‐KD tumors (Fig. S2D,E). Taken together, our results suggest that miR‐223 acts as a critical modulator in cervical cancer development.

### 3.3 E6‐STAT3 signaling regulates miR‐223 transcription

**Fig. 3:**
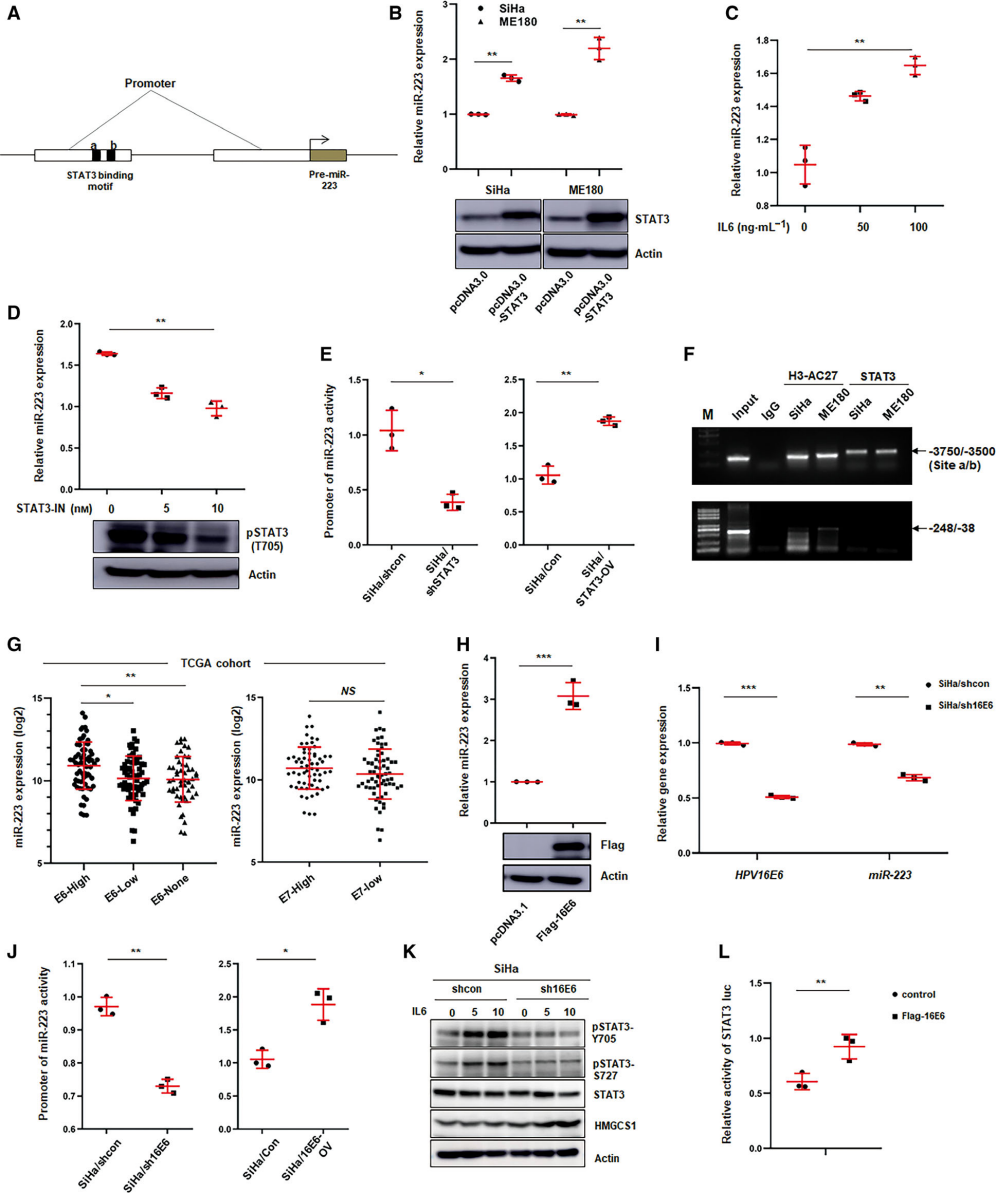
E6 regulates miR‐223 expression through affecting STAT3 activity in CSCC cells. (A) Schematic illustrating the distal and proximal promoters of pre‐miR‐223 gene, and two putative STAT3‐binding motifs on the distal promoter. (B) qPCR analysis of miR‐223 level in CSCC cells transfected with control or STAT3 overexpression plasmids. Blot showed the STAT3 expression in transfected SiHa or ME180 cells. Data represent mean ± SD from three independent experiments. (C–D) miR‐223 level was determined by qPCR in SiHa cells treated with IL‐6 (C) or STAT3 inhibitor (D) at the indicated concentration. Blot showed the level of phosphorylated STAT3 (Tyr 705) in treated cells. Data represent mean ± SD from three independent experiments. (E) Luciferase reporter assay analysis of the activity of miR‐223 promoter in STAT3 knockdown (left) or overexpression (right) and corresponding control cells. Data represent mean ± SD from three independent experiments. (F) ChIP assay analysis of the binding of STAT3 on miR‐223 promoter. The enrichment fragments pulled down by anti‐STAT3 antibody were analyzed by agarose gel electrophoresis. H3‐AC27 (anti‐Acetylated H3K27) served as positive control. These results represent one of three independent experiments. (G) miR‐223 expression was analyzed in indicated groups defined by HPVs E6 or E7 level in TCGA cohort. (H) miR‐223 level was determined by qPCR in E6 overexpression or control cells. The blot showed E6 expression in transiently transfected SiHa cells with pCMV‐Flag‐E6 plasmids by incubated with anti‐Flag antibody. These results represent one of three independent experiments. (I) miR‐223 expression was examined by qPCR in E6 knockdown or control cells. The efficiency of knockdown of E6 was shown as left. (J) Luciferase reporter assay of the activity of miR‐223 promoter in E6 knockdown (left) or overexpression (right) and corresponding control cells. (K) Western blot analysis of phosphorylated STAT3 (Tyr705 and Ser727) in E6 knockdown or control cells treated with IL‐6 as indicated concentration. (L) Luciferase reporter assays of STAT3 activity in 293T cells transiently transfected with control or pCMV‐Flag‐E6 plasmids. Briefly, STAT3‐luc (100 ng·well^−1^) and pRL (50 ng·well^−1^) were cotransfected with control or pCMV‐Flag‐E6 (100 ng·well^−1^) into 293T cells (1 × 10^5^ per well, 48‐well plates), respectively. After 48 h, luciferase activity was determined by dual‐luciferase reporter assay. Data represent mean ± SD from three independent experiments. Data were statistically analyzed with Student's *t*‐test, and value was shown as mean ± SD. *P < 0.05, **P < 0.01, ***P < 0.001.

To further investigate the mechanism underlying aberrant miR‐223 expression in cervical cancer cells, we first analyzed the sequence of the miR‐223 promoter by using jaspar software (Khan *et al*., 2018) and found two conserved binding sites of STAT3 in the distal promoter of the miR‐223 gene (Fig. 3A). To determine whether STAT3 reliably regulated miR‐223 expression, STAT3 was ectopically expressed in CSCC cells, and the miR‐223 level was determined by qPCR. As anticipated, miR‐223 was significantly upregulated in STAT3‐overexpressing cells (Fig. 3B). When cervical cancer cells were treated with IL‐6 which is a major stimulator of STAT3 signaling, the miR‐223 level significantly increased in a dose‐dependent manner (Fig. 3C). In contrast, a STAT3 inhibitor (C188‐9) dramatically suppressed miR‐223 expression (Fig. 3D). Furthermore, to explore whether STAT3 directly regulated miR‐223 expression at the transcriptional level, a luciferase reporter assay was performed. We cloned a fragment from the miR‐223 promoter containing the presumed STAT3 binding site into the pGL4.2 vector (Fig. 3A), which was transfected into STAT3 overexpression or knockdown cells. Indeed, the activity of the promoter was upregulated in STAT3‐overexpressing cells, whereas STAT3 silencing dramatically attenuated the promoter activity (Fig. 3E). STAT3‐luc activity was used as a positive control (Fig. S1B). Additionally, ChIP assays using antibodies against STAT3 were performed in SiHa or ME180 cells to investigate whether STAT3 can recognize and bind to the STAT3‐responsive element. As shown in Fig. 3F, there was robust enrichment of the PCR product in the region near the predicted binging sites (‐3750/‐3500 from the transcriptional start site). The fragment ‐248/‐38 was used as a negative control. These results suggest that STAT3 binds to the promoter of miR‐223 and promotes transcription in cervical carcinoma cells.

The HPV E6 protein plays a critical role in cervical squamous cell transformation by regulating several pathways, including the STAT3 pathway (Morgan *et al*., 2018). Therefore, we wanted to determine whether the E6 protein is involved in STAT3‐mediated miR‐223 expression. First, integrated TCGA analysis was used to investigate the correlation between the E6 levels and miR‐223 levels. Thus, we divided the patients (from the TCGA database) into several groups based on E6 expression or E7 expression, and the miR‐223 levels were compared among these groups. We found that miR‐223 expression was positively correlated with the E6 level, but not with the E7 level (Fig. 3G). To confirm this result, SiHa cells were transiently transfected with E6‐overexpression plasmids, and the miR‐223 levels were determined by using qPCR. Consistent with above observation, E6 overexpression significantly increased the miR‐223 levels (Fig. 3H). In contrast, silencing E6 markedly decreased miR‐223 expression (Fig. 3I). Luciferase reporter assays provided similar results (Fig. 3J). As mentioned above, the E6 protein affects the STAT3 signaling in CSCC cells. To identify this effect in our study, phosphorylated STAT3 was examined in E6‐silenced cells in the context of IL‐6 stimulation. As shown in Fig. 3K, after E6 knockdown, phospho‐STAT3 was obviously decreased. Similar results were obtained from the reporter gene assay (Fig. 3L). Collectively, these data suggest that E6 protein affects miR‐223 expression by regulating the STAT3 signaling pathway.

Taken together, our results strongly suggest that the E6‐STAT3 signaling axis plays a pivotal role in regulating miR‐223 expression in cervical cancer cells.

### 3.4 STAT3 affects cervical cancer development by regulating miR‐223

**Fig. 4:**
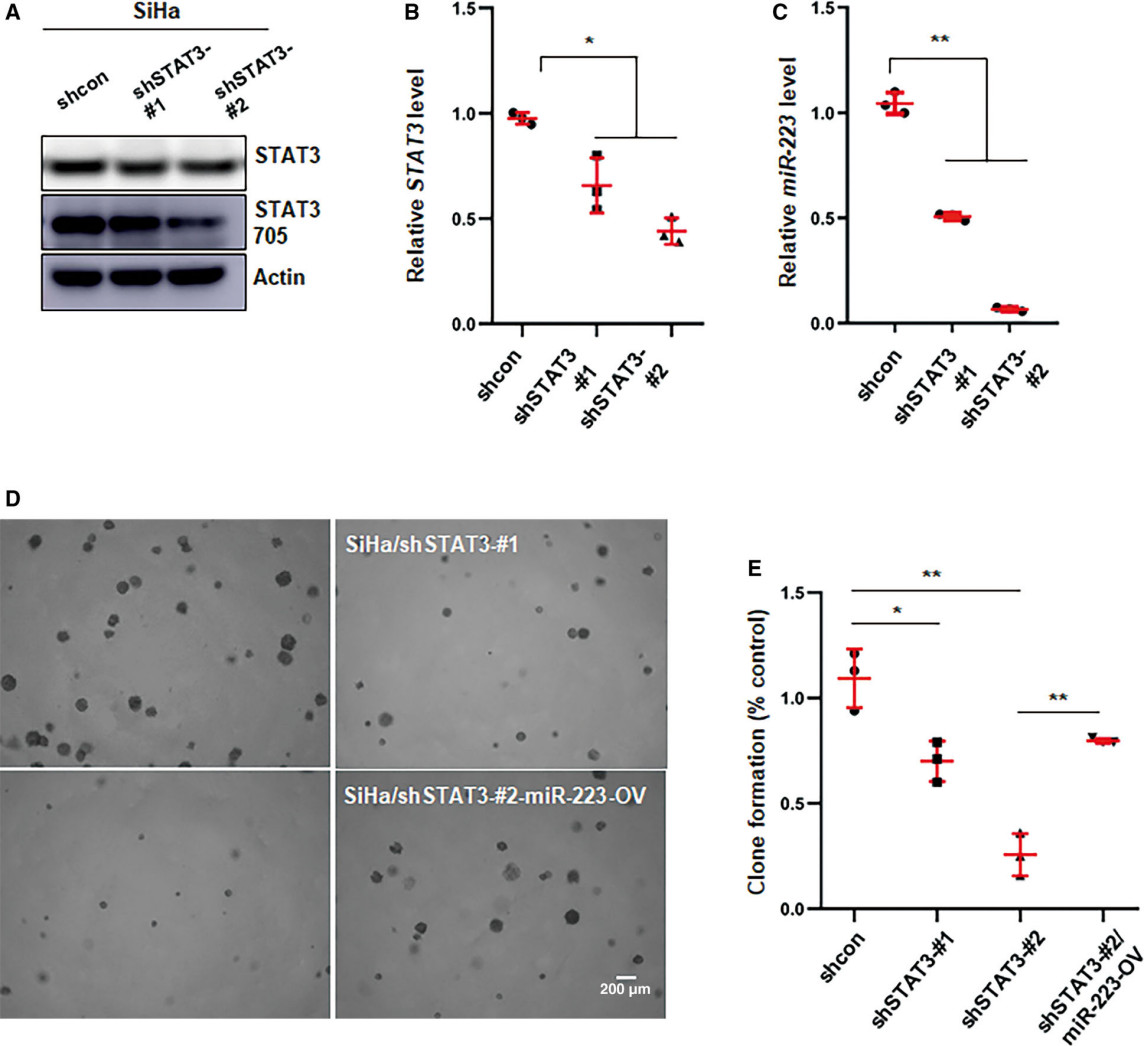
STAT3 promotes cervical cancer progression through regulating miR‐223. (A, B) The efficiency of knockdown of STAT3 was determined by western blot (A) and qPCR (B). (C) qPCR analysis of miR‐223 level in STAT3 knockdown cells. Data represent mean ± SD from three independent experiments. (D) Anchor‐independent growth assay was used to estimate the effect of STAT3 on colony formation of SiHa cells and the rescue of attenuate colony formation in knockdown cells by overexpressing miR‐223. Images represent one of three independent experiments. (E) Shown was statistic result in (D). Images represent one of three independent experiments. Data were shown as mean ± SD from three independent experiments and were statistically analyzed with Student's *t*‐test. *P < 0.05, **P < 0.01.

Based on previous reports, STAT3 is a master regulator of the development of most cancers, including cervical cancer (Johnson *et al*., 2018). To confirm whether STAT3 serves as an important modulator of cervical cancer progression, we established a knockdown cell line by using shRNA that targeted STAT3 transcripts and performed colony formation assays. The efficiency of the knockdown was examined by qPCR and western blotting (Fig. 4A,B). miR‐223 was significantly decreased in STAT3‐KD cells compared with control cells (Fig. 4C). In addition, STAT3‐KD significantly suppressed colony formation, whereas the overexpression of miR‐223 rescued this effect (Fig. 4D,E). In addition, we wanted to investigate whether the regulation of colony formation by E6 protein in CSCC cells was STAT3‐dependent or not. As shown in Fig. S1C, E6 overexpression obviously enhanced the colony formation capacity. However, when treated with STAT3 inhibitor, there was no significant difference between the control group and the E6 overexpression group. These data illustrate that the E6‐STAT3‐miR‐223 axis plays an important in role in cervical cancer development.

### 3.5 TGFBR3 and HMGCS1 are critical downstream targets of miR‐223 in CSCC cells

**Fig. 5:**
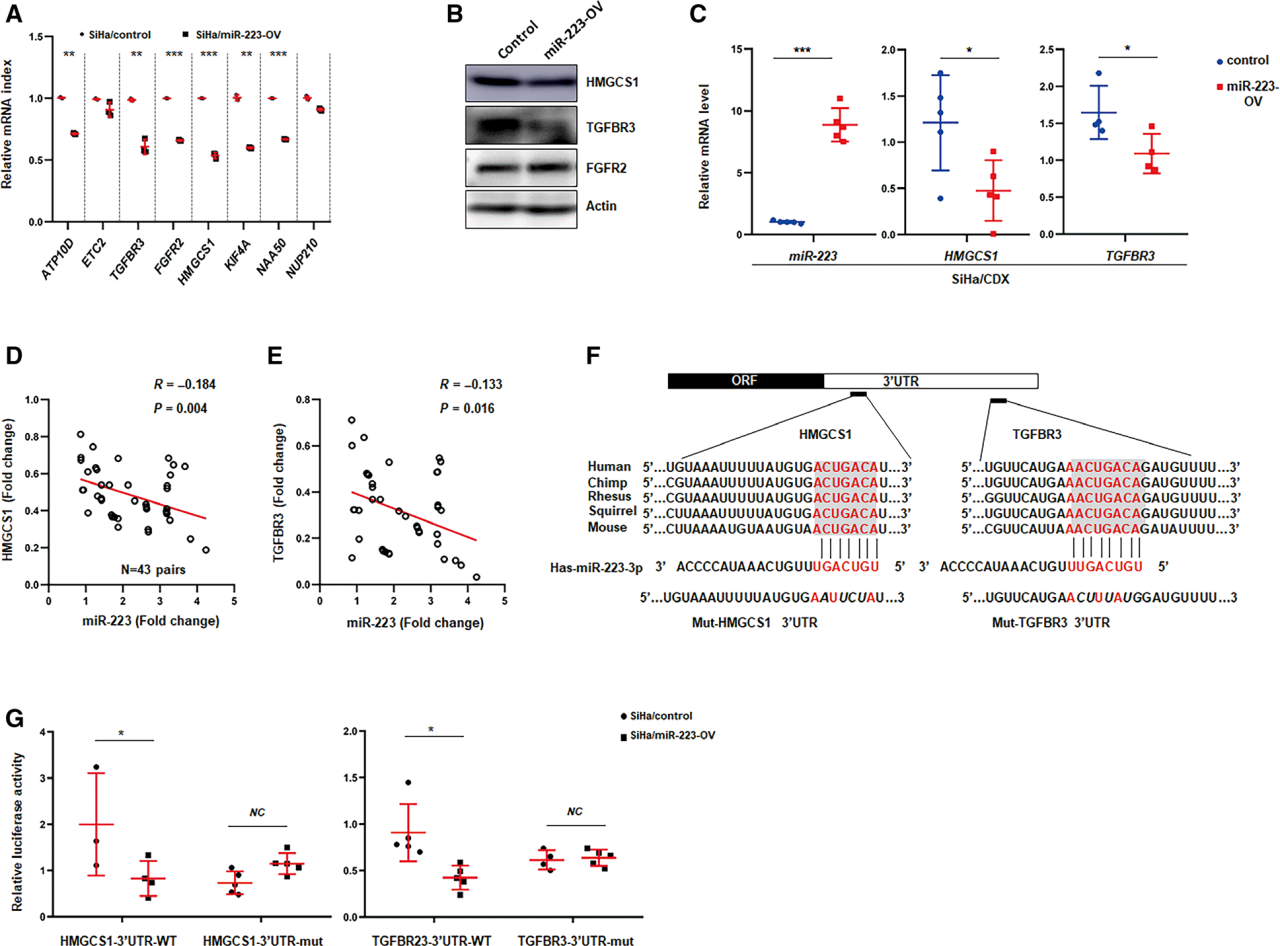
Prediction and validation of TGFBR3 or HMGCS1 as the targets of miR‐223. (A) qPCR analysis of predicated targets of miR‐223 in control or miR‐223 overexpression cells. (B) Western blot analysis of HMGCS1, TGFBR3, or FGFR2 in control or miR‐223 overexpression cells. Actin served as loading control. Blots represent one of three independent experiments. (C) qPCR analysis of the level of TGFBR3 and HMGCS1 of tumors form the implanted mice as indicated. Data represent mean ± SD from three independent experiments. (D, E) Spearman rank correlation scatter plot of the staining score in miR‐223 levels and TGFBR3 levels (D), HMGCS1 levels (E) in CSCC tissues (*n* = 43). (F) Schematic illustrating the highly conserved binding sites for miR‐223 in the 3′ UTR of TGFRB3 and HMGCS1 mRNA and mutation of the indicated nucleotides in seed sequences. (G) Luciferase reporter assays of the effects of the miR‐223 on wild‐type and mutant reporter genes in SiHa cells. Data represent mean ± SD from three independent experiments. *P < 0.05, **P < 0.01, ***P < 0.001.

As a noncoding RNA, microRNA mainly binds to the 3′‐UTR of its targets inducing target mRNA degradation or repressing translation (Macfarlane and Murphy, 2010). To identify the downstream effectors of miR‐223, we first analyzed the integrated data (GSE7803, GSE9750, GSE39001, and GSE63514) to screen the downregulated genes in cervical cancer (Fig. S3A,B). Then, the predicated targets of miR‐223 from TargetScan software were then cross‐validated with the identified downregulated genes from the GEO database (Fig. S3C). To verify these candidates, the transcriptional and protein levels of these predicted targets were examined in genetically edited CSCC cells (Fig. 5A). We found that the TGFBR3 and HMGCS1 levels were significantly downregulated after miR‐223 overexpression (Fig. 5A,B). Consistent with this observation, increased TGFBR3 and HMGCS1 expression was found in miR‐223‐KD tumor tissues recovered from CDX model mice based on IHC assay (Fig. S4A,B). Using qPCR, we also found that TGFBR3 and HMGCS1 were downregulated in miR‐223‐OV tumor tissues (Fig. 5C and Fig. S4C). In addition, TGFBR3 or HMGCS1 expression had a strongly negative correlation with miR‐223 levels in clinical CSCC samples (Fig. 5D,E). These results suggested that miR‐223 suppressed TGFBR3 and HMGCS1 expression in cervical cancer cells. To further validate whether miR‐223 regulated TGFBR3 and HMGCS1 through the presumed binding sites in the 3′‐UTR, a dual‐luciferase reporter assay was conducted. As shown in Fig. 5F, approximately 300‐ or 400‐bp fragment in the 3′‐UTR of TGFBR3 or HMGCS1 mRNA containing the predicted miR‐223 binding sites, respectively, was inserted downstream of the firefly luciferase gene in a pmir‐GLO reporter plasmid. The resulting plasmid was transfected into SiHa cells along with control or miR‐223 overexpression plasmid. As expected, miR‐223 overexpression significantly reduced the luciferase reporter activity compared with the control (Fig. 5G). Consistent with SiHa cells, the decreased activity of the luciferase reporter was also observed in 293T cells that ectopically expressed miR‐223 (Fig. S4D,E). Furthermore, we engineered point mutations into the corresponding binding sites in the 3′‐UTR of TGFBR3 or HMGCS1 to eliminate the putative binding sites (Fig. 5F). The mutated luciferase reporter plasmid exhibited no difference in miR‐223‐overexpressing cells and control cells (Fig. 5G and Fig. S4D,E). Given that the TGFBR3 and HMGCS1 levels were significantly decreased after transfection with miR‐223 in CSCC cells, these reporter assays show that miR‐223 reliably binds with the 3′‐UTR of TGFBR3 or HMGCS1 transcripts repressing their expression at the post‐transcriptional level.

### 3.6 TGFBR3 and HMGCS1 act as repressors in CSCC development

**Fig. 6:**
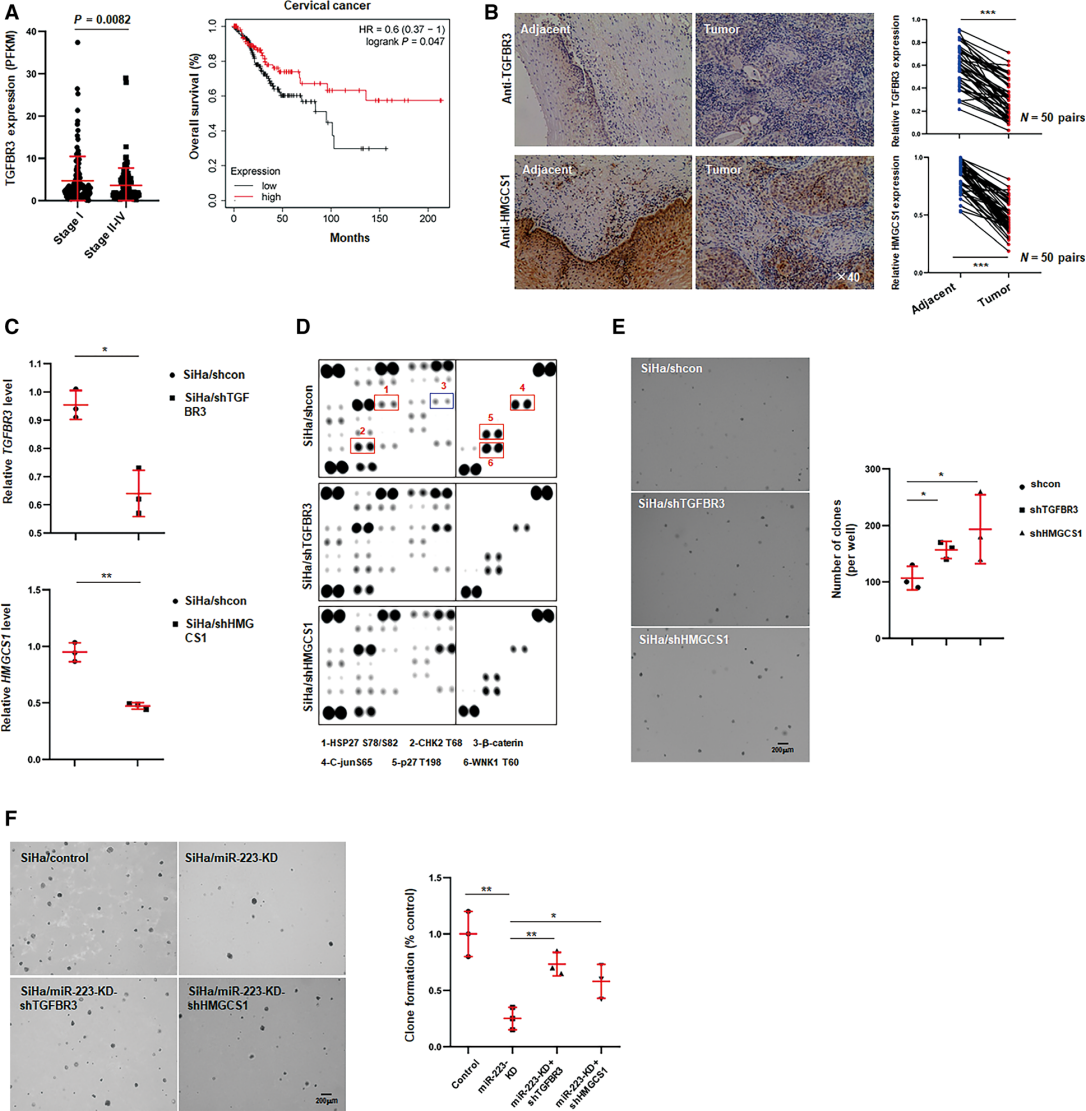
TGFBR3 or HMGCS1 inhibits cervical cancer cell growth and colony formation. (A) Expression value of TGFBR3 was analyzed in indicated stages of CSCC tissues in TCGA cohort, and right was the overall survival rate in TGFBR3‐high or TGFBR3‐low groups. (B) Representative image of IHC staining for TGFBR3 and HMGCS1 of CSCC tissues and matched adjacent tissues (*n* = 50). Mircrographs were shown at original magnifications (×40). IHC score of TGFBR3 and HMGCS1 was shown in right, and the data were shown as the mean ± SD. (C) The efficiency of knockdown of TGFBR3 and HMGCS1 was determined by qPCR in stable transfected cells. (D) Phospho‐kinase assays of the effect of TGFBR3 or HMGCS1 on intrinsic pathways in SiHa cells. (E) Anchor‐independent growth assays of the effect of TGFBR3 and HMGCS1 on colony formation of SiHa cells. Statistic result was shown in right. Data were shown as the mean ± SD. Data represent one of three independent experiments. (F) Representative image of colony formation for double knocking down miR‐223 and TGFBR3 or HMGCS1 in SiHa cells. Statistic result was shown in right. Data were shown as the mean ± SD. *P < 0.05, **P < 0.01, ***P < 0.001.

There are few reports about the role of TGFBR3 and HMGCS1, especially HMGCS1 in cancer progression. As the downstream targets of miR‐223, we hypothesized that TGFBR3 and HMGCS1 might function as repressors in cervical cancer development. To test this hypothesis, we first analyzed the integrated data from TCGA and found that TGFBR3 was expressed at low levels in high‐grade tumor tissues and that high levels of TGFBR3 were associated with favorable prognosis (Fig. 6A). However, HMGCS1 was not significantly different in the defined group as shown in Fig. S3D. Analysis of TGFBR3 and HMGCS1 expression in clinical CSCC samples by IHC assays indicated that TGFBR3 and HMGCS1 were remarkably downregulated in tumor tissues compared with adjacent tissues (Fig. 6B). Next, we established stable transfected cell lines by using lentivirus‐packaged shRNA targeting the coding regions of the TGFBR3 or HMGCS1 transcripts. The knockdown efficiency was determined by qPCR (Fig. 6C). To investigate which intrinsic pathways were affected by TGFBR3 or HMGCS1, we analyzed the status of multiple signaling pathways in the TGFBR3‐KD or HMGCS1‐KD cells by using a phospho‐kinase assay. As shown in Fig. 6D, silencing TGFBR3 or HMGCS1 induced the accumulation of the β‐catenin and decreased the phosphorylation of p27 and CHK2. In addition, knockdown of TGFBR3 or HMGCS1 supported anchorage‐independent growth in CSCC cells (Fig. 6E). To investigate whether miR‐223 promoted the colony formation of CSCC cells by suppressing TGFBR3 or HMGCS1 expression, rescue experiments were performed. As shown in Fig. 6F, knocking down TGFBR3 or HMGCS1 antagonized the effects of miR‐223 silencing on colony formation of CSCC cells. Taken together, these results suggest that TGFBR3 and HMGCS1 are negative regulators of cervical cancer development.

### 3.7 CSCC cell‐derived exosomal miR‐223 induced elevated IL‐6 levels in the coculture system

**Fig. 7:**
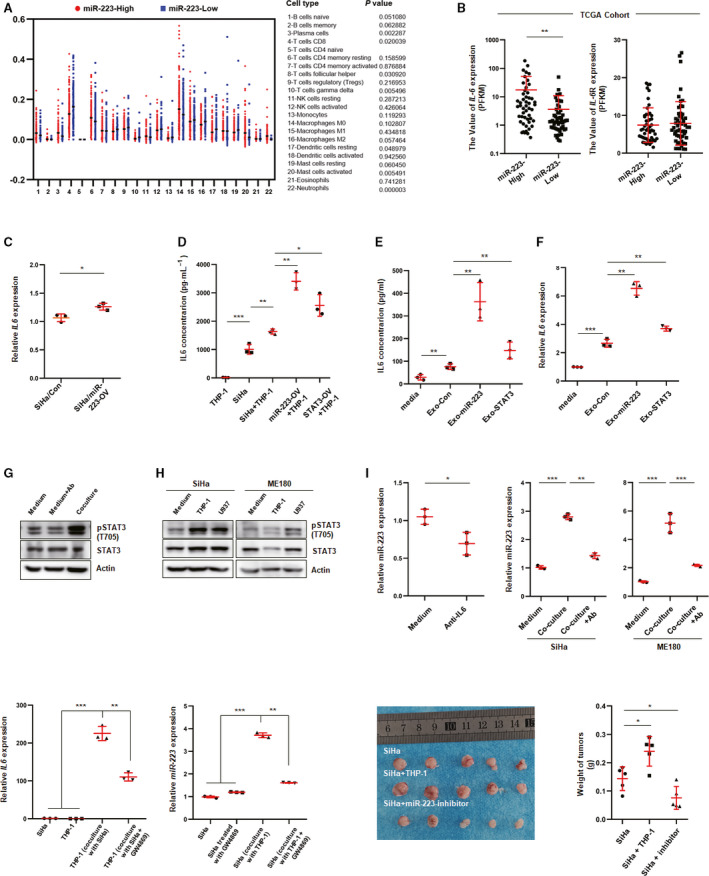
Exosomal miR‐223 promotes IL‐6 expression in monocyte/macrophage. (A) The proportion of immune cells was analyzed by CIBERSORT software based on the RNAseq data in TCGA cohort. (B) Value of IL‐6 and IL‐6R expression in miR‐223‐high or miR‐223‐low group in TCGA cohort. (C) IL‐6 level was determined in miR‐223‐OV or control cells by using qPCR. (D) IL‐6 level was determined by ELISA in SiHa culture, THP‐1 culture, or coculture as indicated. Data represent one experiment out of *n* = 3 performed in triplicates. (E, F) IL‐6 expression was examined by ELISA or qPCR in exosome‐treated macrophage derived from THP‐1 (10 μg·mL^−1^ PMA for 24 h). Induced macrophage was treated with exosomes for 24 h, and LPS (100 ng·mL^−1^) was added into the culture for another 24 h. Then, the RNA extracted from treated cells was reversely transcribed into cDNA for examining gene expression by using specific primers. (G, H) Phosphorylated STAT3 was examined by western blot assay using anti‐STAT3 (Try 705)‐specific antibody in SiHa cells which were cocultured with or without THP‐1 or U937 cells. Blots represent one of three independent experiments. (I) miR‐223 expression was examined by qPCR in IL‐6‐neutralizing antibody (50 ng·mL^−1^)‐treated SiHa cells which were replaced with condition medium from THP‐1 culture simultaneously. (J) qPCR analysis of miR‐223 level in SiHa treated with or without anti‐IL‐6 antibody (50 ng·mL^−1^) for 24 h (left), or in SiHa cells cocultured with or without THP‐1 which treated with or without anti‐IL‐6 antibody (right). Data were shown as mean ± SD. (K) IL‐6 was determined by qPCR in SiHa which was cocultured with THP‐1 treated with or without exosome inhibitor (GW4869 10 μm). (L) miR‐223 expression was examined in THP‐1, which was cocultured with or without SiHa, treated with or without exosome inhibitor. (M) Representative images of tumors from each group. (N) Quantitative analysis of the tumor weights in (M) *P < 0.05, **P < 0.01, ***P < 0.001.

The tumor microenvironment consists of several kinds of cells, such as immune cells, stromal cells, and tumor cells, and a complex cross‐talk occurs among these cells. It is well accepted that tumor cell‐derived cytokines or exosomes skew immune cells toward to an alternative activation status, which in turn secrete many pro‐inflammatory factors, promoting cancer development and metastasis (Kim and Bae, 2016; Quail and Joyce, 2013). To investigate whether cervical cancer cells had an important influence on the tumor microenvironment, integrated data (TCGA) were used to analyze the components of immune cells in the CESC samples by using CIBERSORT software, which was developed by Newman *et al*. (2015). As shown in Fig. 7A, the proportion of CD8^+^ T cells was significantly decreased in the miR‐223‐high cluster. Furthermore, pro‐inflammatory IL‐6 which has been reported to be a critical modulator of cancer progression was significantly upregulated in the miR‐223‐high group, whereas the IL‐6R level was not different between miR‐223‐high and miR‐223‐low groups (Fig. 7B). Although miR‐223 enhanced IL‐6 expression in CSCC cells to some extent, the difference in the IL‐6 levels exhibited in the tumor tissues (TCGA cohort) was considered to be contributed by other cells (Fig. 7C). The IL‐6 concentration in coculture was significantly higher than that in the THP‐1 or SiHa individual cultures (Fig. 7D). miR‐223 or STAT3 overexpression clearly increased IL‐6 levels in the coculture system. These results suggested that the interaction between CSCC cells and monocytes promotes IL‐6 production. It is well known that exosomes play a critical role in intercellular communication in microenvironment. Here, we isolated exosomes from the conditioned medium of CSCC cells and added them to the culture of THP‐1‐derived macrophages (treated with phorbol 12‐myristate 13‐acetate for 24 h) to investigate the IL‐6 expression. As shown in Fig. 7E,F, IL‐6 was obviously increased in exosome‐treated cells, especially in miR‐223‐OV or STAT3‐OV exosome‐treated cells. On the other hand, coculture with SiHa and THP‐1 cells induced a dramatic increase in phosphor‐STAT3 in SiHa cells, whereas the IL‐6‐neutralizing antibody attenuated this effect (Fig. 7G). Similar results were observed in the ME180‐U937 coculture system (Fig. 7H). As mentioned in Fig. 3C, IL‐6 treatment induced the miR‐223 expression in SiHa cells. Consistent with this observation, IL‐6‐neutralizing antibodies decreased the miR‐223 levels in SiHa cells that were treated with conditioned medium from THP‐1 cells (Fig. 7I). Consistently, in the coculture system, the expression of miR‐223 in SiHa cells was obviously upregulated, whereas the IL‐6‐neutralizing antibody eliminated this effect (Fig. 7J). Based on these findings, it is rational to conclude that cross‐talking mediated by exosomes between CSCC cells and monocytes induces IL‐6 expression, which promotes STAT3 activation and results in miR‐223 upregulation. In addition, GW4869 (exosome inhibitor) inhibited IL‐6 expression of THP‐1 cells (Fig. 7K) and miR‐223 expression of SiHa cells (Fig. 7L) in the coculture system. Furthermore, by using the CDX model, we found that THP‐1 significantly promotes tumor growth (Fig. 7M,N). To investigate whether miR‐223 could serve as therapeutic target for cervical cancer, cholesterol‐conjugated miR‐223 inhibitors or controls were delivered *via* the tail vein every 4 days for 16 days after implantation. As shown in Fig. 7M,N, the tumor sizes and weights were dramatically decreased after inhibitor treatment compared with those of the miRNA‐NC treatment group. Overall, these data suggest that the interaction between CSCC cells and monocytes/macrophages is critical for cancer development.

## 4 Discussion

**Fig. 8:**
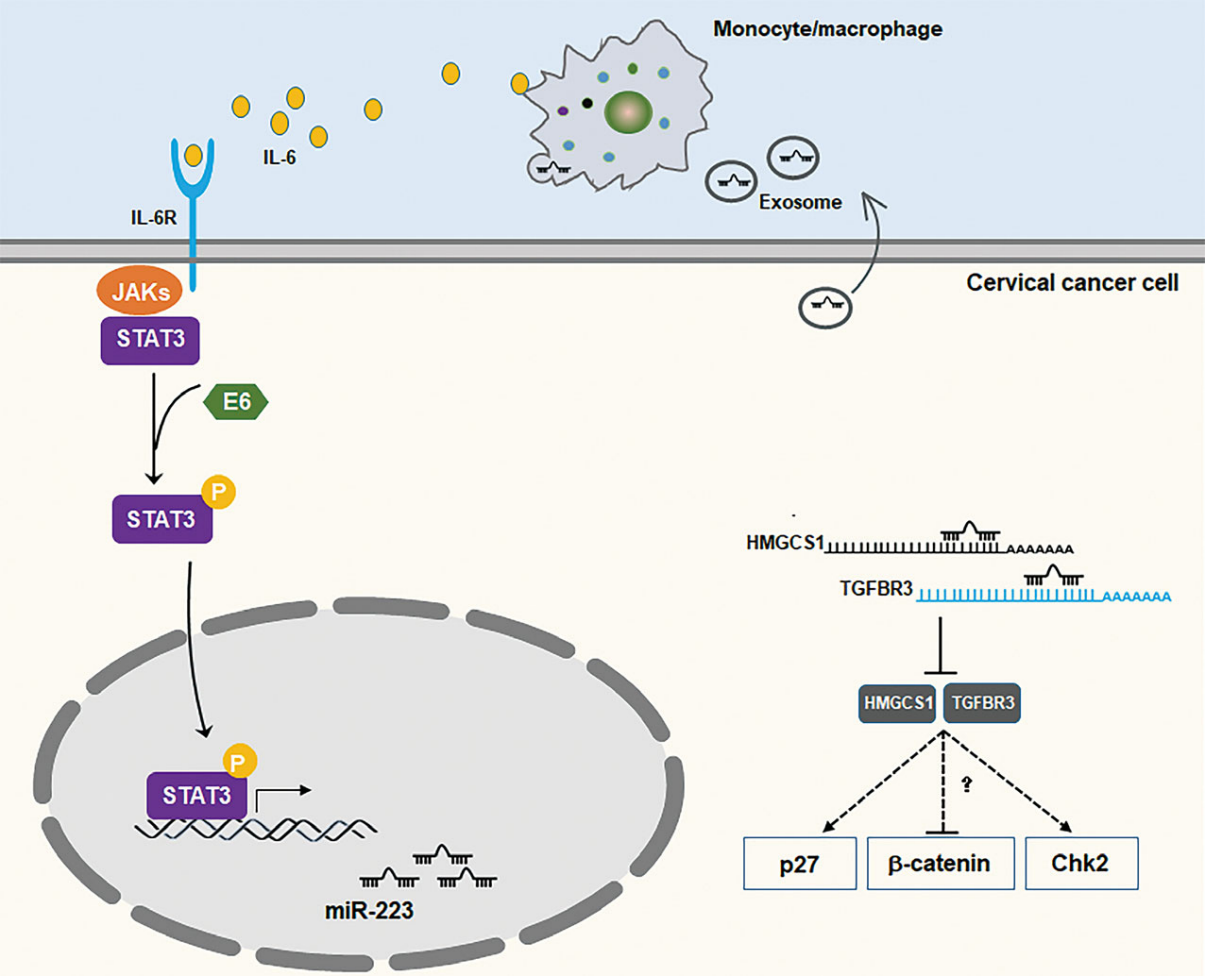
The schematic model for STAT3‐miR‐223‐TGFBR3/HMGCS1 axis in CSCC development. In CSCC cells, IL‐6‐induced STAT3 activation is enforced by HPVs E6 protein. Upregulated phosphorylation of STAT3 enters into nuclear to bind on the promoter of miR‐223 and accelerates its expression. Increased miR‐223 inhibits TGFBR3 and HMGCS1 expression through promoting TGFBR3 or HMGCS1 mRNA degradation in cytoplasm. Downregulated TGFBR3 or HMGCS1 lose its effect on inhibiting β‐catenin accumulation or promoting phosphorylation of p27 and CHK2. In microenvironment, exosomal miR‐223 induces IL‐6 expression in monocyte/macrophage promoting STAT3 activation in CSCC cells.

In the present study, we first demonstrated the oncogenic role of miR‐223 in CSCC carcinogenesis. Specifically, we revealed that miR‐223 was highly expressed in cervical cancer and effectively promoted the migration and tumorigenesis of CSCC cells in both *in vitro* and *in vivo* models. Mechanistically, we proved that miR‐223 bound to the mRNA of TGFBR3 and HMGCS1 and suppressed their expression at the post‐transcriptional level. TGFBR3 and HMGCS1 acted as repressors of cervical cancer development, attenuating the activity of colony formation *in vitro*. Furthermore, we demonstrated that the increased expression of miR‐223 was mediated by the transcription factor STAT3 which was regulated by the HPV E6 protein. In addition, we showed that CSCC cells exhibited exosomal miR‐223‐induced IL‐6 secretion, and in turn, IL‐6 promoted STAT3 activation in CSCC cells. Our data describe the regulation and mechanism of the effect of miR‐223 on TGFBR3 and HMGCS1 and the possibility that the STAT3‐miR‐223‐TGFBR3/HMGCS1 axis could act as a potential therapeutic target for CSCC (Fig. 8).

miR‐223 has mostly been reported to be critical for hematopoiesis (Chen *et al*., 2004). In our previous work, we demonstrated that miR‐223 promotes M2 polarization by targeting MOZ (Jiang *et al*., 2019). Interestingly, miR‐223 has been reported to act as a repressor in AML progression by targeting the transcription factor E2F1 (Pulikkan *et al*., 2010). In solid tumors, miR‐223 functions as a tumor‐suppressive miRNA or positive regulator in different cancer types. In pancreatic cancer cells, miR‐223 promotes cancer cell proliferation and invasion by targeting PDS5B which acts as a repressor of pancreatic cancer development (Ma *et al*., 2019). In breast cancer, miR‐223 functions as a repressor in the initiation or chemoresistance of breast cancer (Citron *et al*., 2020; Masciarelli *et al*., 2014; Pinatel *et al*., 2014). Particularly, loss of miR‐223 is an early event during breast transformation (Citron *et al*., 2020), and stroma derived miR‐223 inhibits tumor cell migration and invasion (Pinatel *et al*., 2014). These studies suggest that the miR‐223/E2F1 axis plays an important role not only in blood cancer, but also in solid cancers. In another report, exosomal miR‐223 plays a unique role in the cross‐talk between macrophages and ovarian cancer cells in the context of chemotherapy resistance (Zhu *et al*., 2019). However, in lung squamous cell carcinoma, miR‐223 functions as a tumor suppressor (Luo *et al*., 2019). In hepatocellular carcinoma cells, miR‐223 is found to target Rab1, which is critical for mTOR pathway, and to promote apoptosis (Dong *et al*., 2017). Interestingly, a report indicates that miR‐223 suppresses cell proliferation by targeting IGF‐1R in HeLa cells, which models cervical adenocarcinoma (Jia *et al*., 2011). In contrast to that report, our results strongly suggest that miR‐223 positively regulates tumorgenesis in SiHa and ME180 cells, which are cervical squamous carcinoma cells. This discrepancy can be explained by the completely different intrinsic mechanisms underlying the tumor initiation and development of the different cell types. Convincingly, published reports indicate that miR‐223 is highly expressed in cervical cancer tissues or raft tissues derived from human keratinocytes transfected with HPV 18 DNA (Wang *et al*., 2008). Furthermore, in our present work, we identified two novel targets of miR‐223, namely TGFBR3 and HMGCS1. Although few studies have discussed the role of these two proteins in tumorigenesis, these proteins have been found to be downregulated in cervical cancer (Kori and Yalcin Arga, 2018). Similarly, our data indicate that TGFBR3 and HMGCS1 function as repressors of tumorgenesis in CSCC. In addition, accumulated β‐catenin and downregulated phosphorylated level of p27 and CHK2 were found in TGFBR3 or HMGCS1 knockdown cells by using a phospho‐kinase array. Wnt/β‐catenin pathway contributes greatly to cancer development, and phosphorylated p27 and CHK2 are well‐known inhibitors in cancer cells. In light of these current studies, we proposed that TGFBR3 or HMGCS1 suppressed cervical cancer progression through negatively regulating the Wnt/β‐catenin pathway or positively promoting phosphorylation of p27 and CHK2, at least partially. However, the detailed mechanisms underlying the regulation of these pathways by TGFBR3 or HMGCS1 need to be explored in the future.

As a transcription factor, STAT3 enters into the nucleus after being phosphorylated to bind target promoters to regulate their expression and is involved in numerous important biological processes (Yu *et al*., 2014; Yu *et al*., 2009). Over 70% of cancers are associated with hyperactivated STAT3. In cervical cancer, several reports show that STAT3 is critical for cell transformation and cycle progression (Takemoto *et al*., 2009). In our studies, we found that phosphorylated STAT3 was increased in cancer tissues, as compared with adjacent tissues (data not shown). In our study, we proved that STAT3 upregulated miR‐223 expression by directly binding to the promoter of miR‐223. And, the E6 protein plays a pivotal role in IL‐6‐stimulated STAT3 activation, suggesting E6 protein induced miR‐223 upregulation in CSCC cells by affecting STAT3 activation, at least partially. However, the detailed mechanism underlying the regulation of STAT3 activation by E6 remains to be determined in future works.

## 5 Conclusion

Taken together, our finding provides the new STAT3‐miR‐223‐TGFBR3/HMGCS1 axis in cervical cancer development. Mechanistically, activated STAT3 mediated by HPVs E6 protein binds to the promoter of miR‐223 gene increasing its transcription, which binds to the 3′‐UTR of TGFBR3 or HMGCS1 mRNA repressing their expression. In other hand, exosomal miR‐223 derived from cervical cancer cells induces IL‐6 secretion from monocyte/macrophage, which, in turn, activates STAT3 signals in cervical cancer cells. However, more in‐depth investigations of the role of TGFBR3 or HMGCS1 in cervical cancer progression are required in future. Moreover, the mechanism of exosomal miR‐223 regulating IL‐6 expression in monocyte/macrophage should be uncovered in future works.

## Conflict of interest

The authors declare no conflict of interest.

## Author contributions

JZ performed the experiment, analyzed the data, and prepared the figures. MJ designed the experiments, performed the experiments, analyzed the data, wrote the manuscript, and supervised the research. LQ performed the experiments and collected clinical specimen. XL performed the experiments. WS performed the experiments. YG discussed the manuscript. YZ discussed the manuscript.

## Supporting information


**Fig. S1.** E6‐STAT3 promotes colony formation.
**Fig. S2.** miR‐223 promotes CSCC tumorgenesis.
**Fig. S3.** Screening the putative targets of miR‐223 in CSCC.
**Fig. S4.** TGFBR3 or HMGCS1 expression is suppressed by miR‐223.
**Table S1.** Primer sequences used in reverse transcription quantitative polymerase chain reaction.
