# Supplementary material for: The STAT3‐miR‐223‐TGFBR3/HMGCS1 axis modulates the progression of cervical carcinoma
Source: Mol Oncol. 2020 Jul 1;14(9):2313–31. doi: 10.1002/1878-0261.12737 (PMC7463355; doi:10.1002/1878-0261.12737)

**Supplement figures and table**

**Fig. S1. E6-STAT3 promotes clone formation.**

(A) miR-223 expression was determined by QPCR in sphere cells and parental control cells. Actin served as reference gene. (B) Luciferase reporter assay of STAT3 activity in STAT3 knockdown or overexpression cells and corresponding control cells. (C) Representative images of clone formation of HPV E6 overexpression cells and control cells treated with or without STAT3 inhibitor (C188-9). Right was the statistics analysis of left. Data were shown as the mean ±SD. **P*<0.05, ***P*<0.01, ****P*<0.001.

**Fig. S2. miR-223 promotes CSCC tumorgenesis.**

(A) Schematic overview regarding the construction of miR-223 overexpression and knockdown in CSCC cells. (B) The efficiency of miR-223 overexpression or knockdown in SiHa cells was determined by QPCR. The relative miRNA expression was calculated with the equation 2^-∆∆CT^, where ∆CT= (CT_miR-223_-CT_U6_), ∆∆CT = ∆CT_miR-223-OV/KD_-∆CT_control_). (C) Representative images of tumor-bearing nude mice of each group. (D) Representative images of H&E staining and IHC staining for Ki67 and VEGFA of tumors from the implanted mice. Right was statistics analysis of left. (E) Representative images of IHC staining for Ki67 of tumors from the indicated implanted mice Right was statistics analysis of left. (F) Representative images of tumors from implanted mice. Right was quantitative analysis of tumor weight of left. Data were shown as the mean ±SD. **P*<0.05, ***P*<0.01, ****P*<0.001.

**Fig. S3. Screening the putative targets of miR-223 in CSCC.**

(A) Volcano plots of upregulated or downregulated genes in CSCC tissues in GSE7803, GSE9750, GES39001 and GSE63514 cohort. (B) Venn diagram analysis of downregulated genes in (A). (C) Venn diagram analysis of downregulated genes and predicated genes of miR-223 generated by Targetscan software. (D) The value of HMGCS1 was analyzed in defined stages of CSCC in TCGA cohort.

**Fig. S4. TGFBR3 or HMGCS1 expression is suppressed by miR-223.**

(A-B) Representative images of IHC staining for TGFBR3 (A) or HMGCS1 (B) in tumors from implanted mice. Right was statistics analysis of staining score in left. (C) HMGCS1 and TGFBR3 levels were examined by QPCR in tumors from implanted mice. (D-E) Luciferase reporter assay of 3’UTR of HMGCS1 or TGFBR3 mRNA in 293T cells transfected with or without miR-223 overexpression plasmids. Data were shown as the mean ±SD. **P*<0.05, ***P*<0.01, ****P*<0.001, *NS* = not significant.

**Table S1.** Primer sequences used in reverse transcription quantitative polymerase chain reaction.


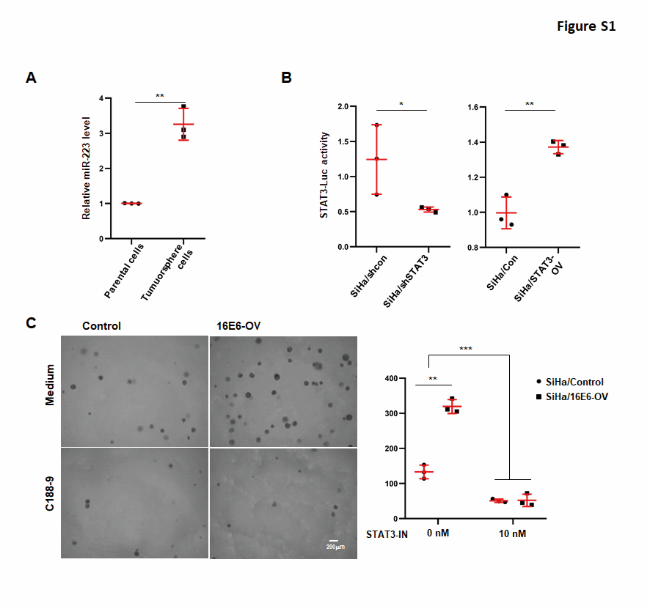


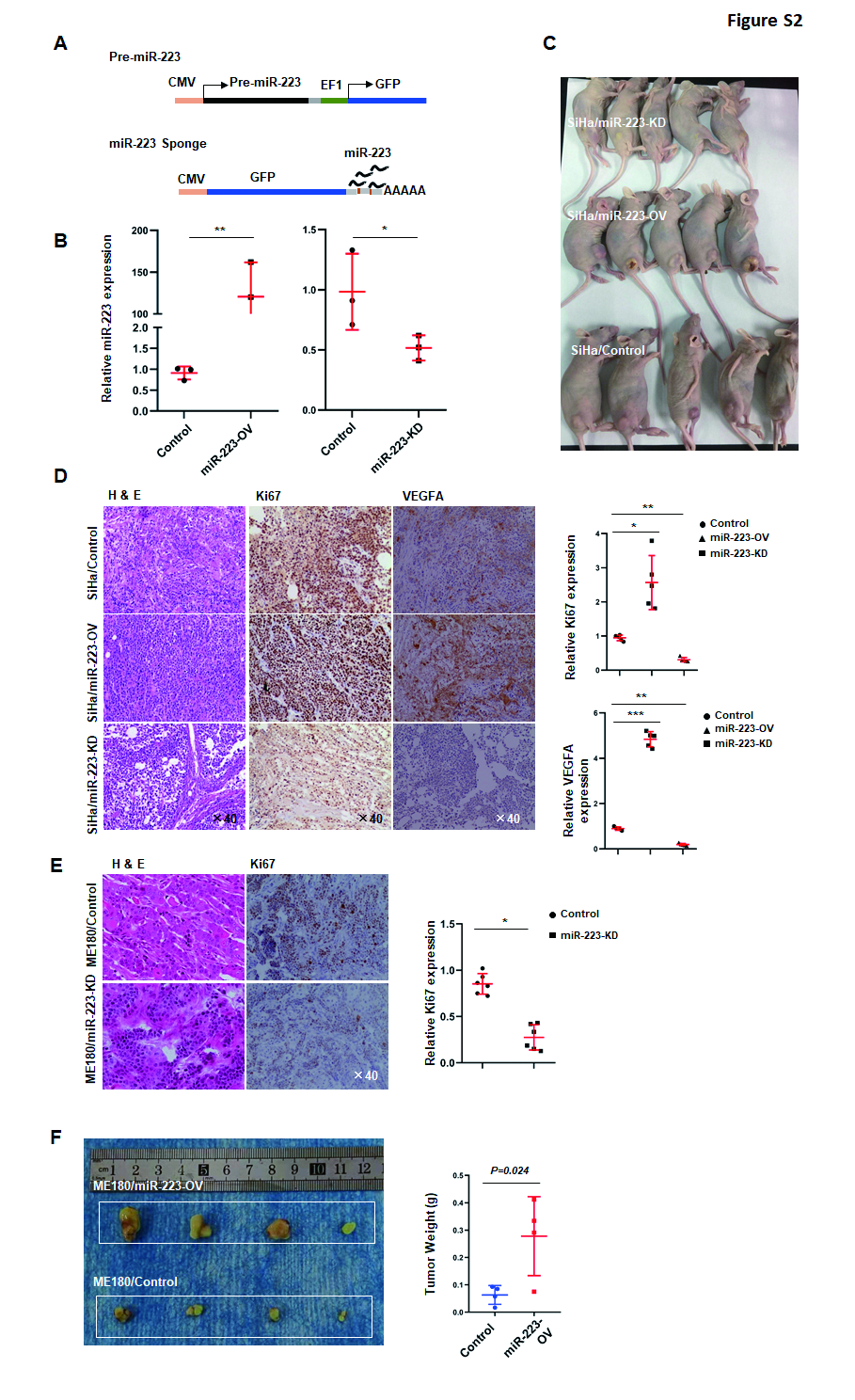


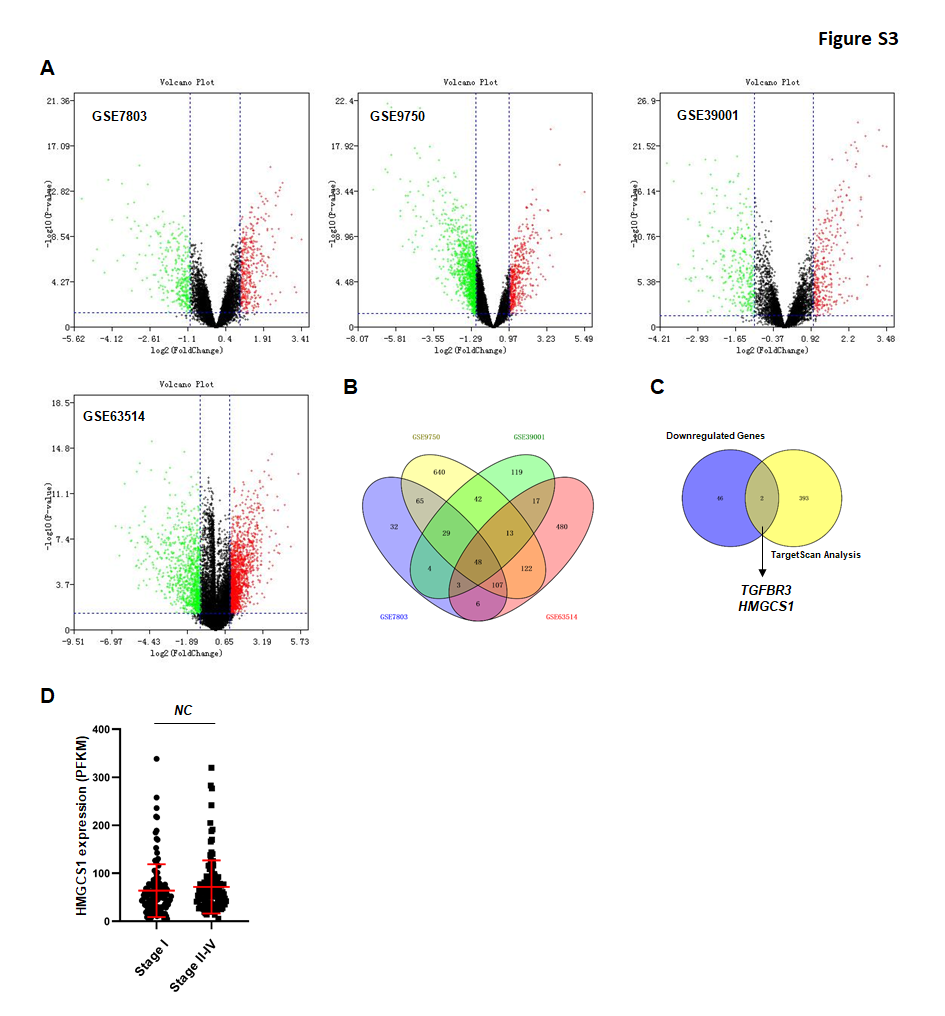


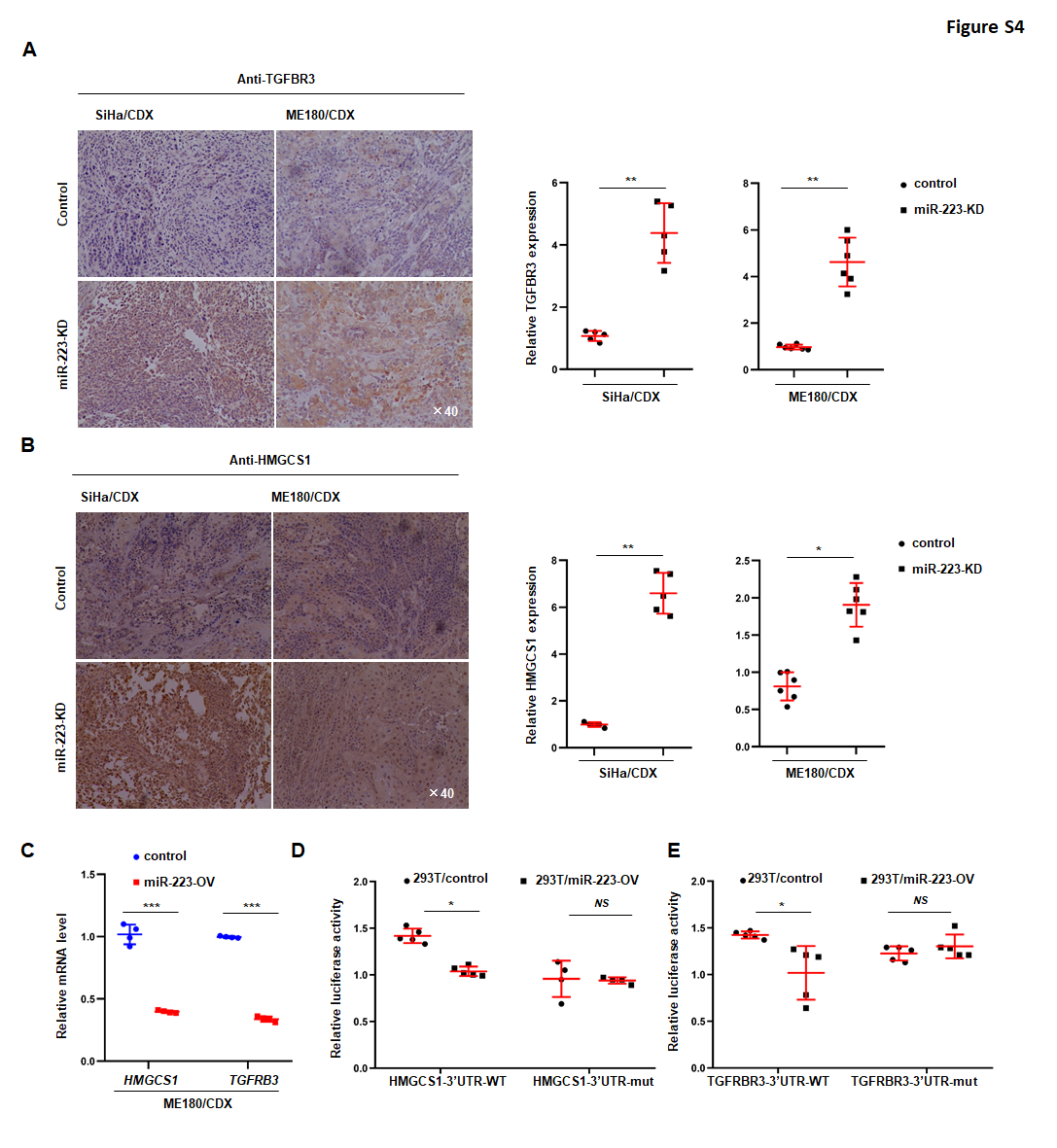


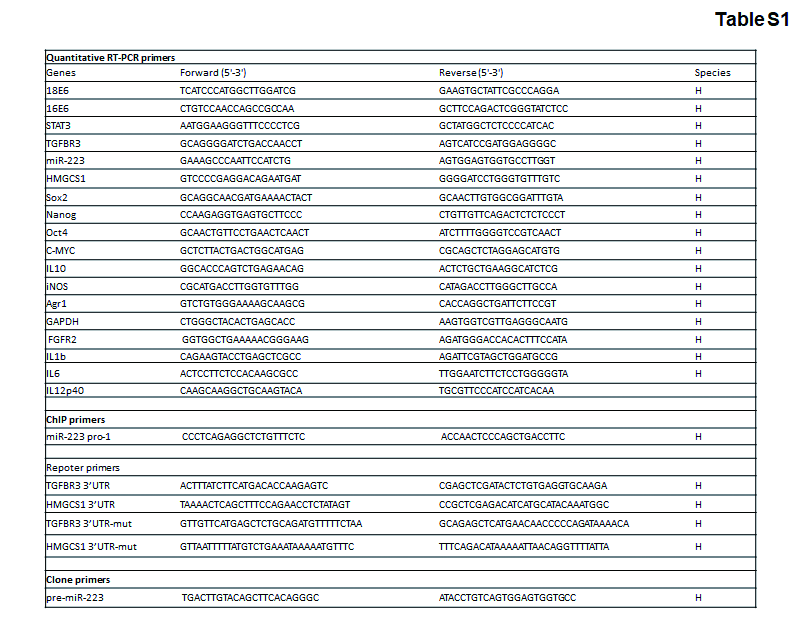

Supplement: Supplementary file 1 — Fig. S1. E6‐STAT3 promotes colony formation. Fig. S2. miR‐223 promotes CSCC tumorgenesis. Fig. S3. Screening the putative targets of miR‐223 in CSCC. Fig. S4. TGFBR3 or HMGCS1 expression is suppressed by miR‐223. Table S1. Primer sequences used in reverse transcription quantitative polymerase chain reaction. [file MOL2-14-2313-s001.docx]
